# Supplementary figures and images for: Transfer learning enables prediction of CYP2D6 haplotype function
Source: PLoS Comput Biol. 2020 Nov 2;16(11):e1008399. doi: 10.1371/journal.pcbi.1008399 (PMC7660895; doi:10.1371/journal.pcbi.1008399)

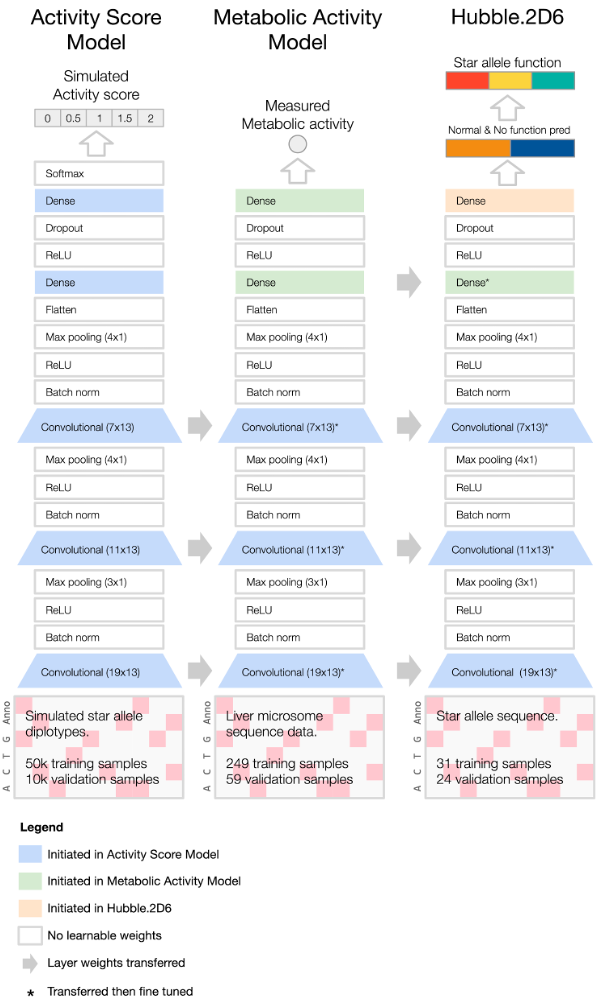

Supplement: S1 Fig — Here we show the architecture of each model that contributes to the final model as well as the logic of the learned weight transfers between models. We train three models total, including two pre-training steps and a final model that predicts star allele function (Hubble.2D6). The first model predicts the CYP2D6 Activity Score for simulated diplotype sequence data (a classification problem). The second predicts the measured metabolic activity for liver microsome data (regression). The final model predicts categorical function of a star allele (classification). Layer colors represent which model the learned weights were initialized in. The numbers in parentheses (e.g. 19x13) represent the dimensions of the filter for that layer. Layers with colors indicate layers with learnable weights, colors indicate which training step the weights were learned in. Asterisks indicate layer weights were transferred then fine-tuned in the corresponding model. (PNG) [file pcbi.1008399.s001.png]

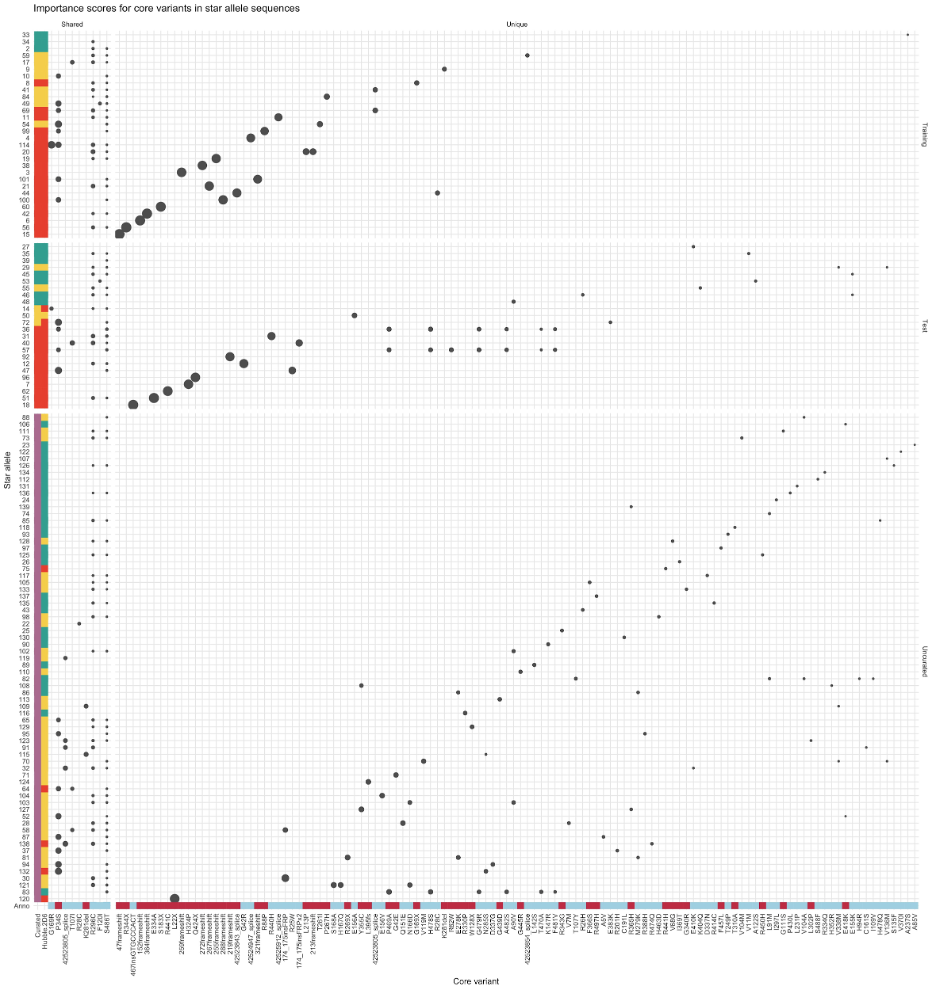

Supplement: S2 Fig — Star alleles are along the y-axis and core variants (both amino acid changes and non-coding changes) are listed along the x-axis. Each dot represents the importance of the core variant to the final prediction as determined by DeepLIFT. The size of the dot represents the value of the importance score, with larger dots indicating variants with larger importance scores, typically associated with a negative impact on function. Star alleles are annotated with the curated function as well as the Hubble.2D6 predicted function. Star alleles are divided along the y-axis between star alleles that were included in the training data (top) and those used as test samples (bottom). Star alleles are sorted by the sum of the importance scores, with those with the largest sums at the bottom. Core variants are divided along the x-axis by those that are uniquely in either the training or test samples (right), and those that are shared between star alleles in train and test (left). Core variants are sorted by their mean importance score across all star alleles. Core variants are annotated with the deleteriousness annotation used in the functional variant representation. (PNG) [file pcbi.1008399.s002.png]
